# Genomic landscape of a long-term surviving patient with metastatic cancer of the larynx: a case report

**DOI:** 10.3389/fonc.2025.1691802

**Published:** 2025-12-02

**Authors:** Vesna Bišof, Majana Soče, Anita Škrtić, Sven Seiwert, Marijana Peričić Salihović

**Affiliations:** 1 Department of Oncology, University Hospital Center Zagreb, School of Medicine, University of Zagreb, Zagreb, Croatia; 2 Department of Pathology, School of Medicine, University of Zagreb, Zagreb, Croatia; 3 Department for Fundamental Interdisciplinary Research Institute for Anthropological Research, Zagreb, Croatia

**Keywords:** laryngeal squamous cell carcinoma, recurrent-metastatic, case-report, long-term survival, genomic profiling

## Abstract

Head and neck squamous cell carcinoma (HNSCC) remains a major global health challenge, with limited long-term survival in the recurrent/metastatic (R/M) setting. Here, we present a rare case of a patient with metastatic laryngeal squamous cell carcinoma who survived eight years after diagnosis of metastatic disease, treated only with standard chemotherapy, metastasectomy, and palliative radiotherapy. To explore potential genomic underpinnings of this unusually favorable outcome, we performed next-generation sequencing of tumor (lymph node and lung metastases) and germline DNA. Mutations were analyzed across ten key oncogenic signaling pathways and compared with the COSMIC and OncoKB databases. Somatic mutations were found in all ten pathways, with multiple 3′ UTR variants in *EIF4EBP1* and *CTBP2*, genes implicated in translational regulation and transcriptional repression, respectively. Germline analysis revealed 21 high-impact mutations, three of which were rare and potentially deleterious. Our findings suggest that subtle regulatory variants may contribute to favorable clinical outcomes in rare long-term survivors of R/M HNSCC. Further studies are needed to identify predictive biomarkers in this unique subgroup of patients.

## Introduction

Head and neck cancer (HNC) ranks as the seventh most common cancer globally, accounting for more than 890,000 new cases and 450,000 deaths annually (1). Approximately 90% of HNCs are squamous cell carcinoma (SCC), that arise from the epithelial lining of the oral cavity, pharynx and larynx (2). Laryngeal cancer accounts for almost 25-30% of all HNCs. At diagnosis, nearly half of patients present with locoregionally advanced disease, and approximately 50% will experience relapse after primary treatment with distant metastases and/or local or regional disease (3). Synchronous distant metastases occur in up to 15% of patients (3), with the lungs being the most common site, accounting for two-thirds of all distant metastases (4).

The prognosis of patients with recurrent or metastatic (R/M) head and neck squamous cell cancer (HNSCC) is generally poor. Surgery and/or radiotherapy is possible in 20% of patients while the majority of them are treated with systemic treatment. Since 2008, the addition of cetuximab to platinum based chemotherapy in the first line therapy as well as the implementation of pembrolizumab and nivolumab in the second line therapy, and finally the use of pembrolizumab alone or in combination with chemotherapy in programmed death-ligand 1 (PD-L1) positive tumors in the first line therapy have improved the survival of these patients (5–8). In spite of that, the best median survival is no longer than 15 months depending on patient- and tumor- related factors. Furthermore, spatial and temporal tumor heterogeneity has been well recognized (9). New effective treatment options are needed to further improve treatment outcomes.

Nonetheless, rare long-term survivors exist even among patients treated with chemotherapy alone. In the control arms of the EXTREME (5) and ECOG-ACRIN E1305 (10) trials, a small proportion of patients (0.9% and 1.5%, respectively) survived five years or longer. Although the biology of their disease already proved the aggressiveness by the formation of metastases and relapses they had somehow better outcome than other patients. These outliers raise the question: is their survival merely an anomaly, or do they represent a unique prognostic subgroup of R/M HNSCC patients who may benefit from less intensive treatment? If so, how to recognize them?

Here, we report the case of a patient with metastatic laryngeal SCC who survived for eight years following diagnosis of metastatic disease, despite receiving only standard chemotherapy without targeted therapy or immunotherapy. We performed genomic profiling on lymph node and lung metastases, along with germline DNA analysis, aligning our findings with cancer-associated genes catalogued in the COSMIC (11) and OncoKB (12, 13) databases. Additionally, mutations were mapped onto ten key oncogenic signaling pathways (14).

## Materials and methods

Germline DNA was extracted from whole blood using Nucleon spin blood kit (Merchey-Nagel). DNA from paraffin was extracted using QIAamp DNA FFPE Tissue Kit (Qiagen). The purity of the FFPE samples was assessed by a pathologist from H&E-stained slides and both samples were of sufficient quality. Whole genome sequencing was performed in commercial facility as follows: libraries were constructed using Illumina TruSeq DNA PCR-Free kit and TruSeq Nano DNA Sample Preparation Kit. Sequencing was performed on Illumina HiSeq sequencing system. The raw images and base calling were generated on Illumina Platform. Phred quality scores were as follows genomic DNA: Q20: 97.8%, Q30: 96.5%; lung metastasis: Q20: 97.5%, Q30: 94.0%; lymph node: Q20: 96.7%, Q30: 92.2%. Coverage depth metrics revealed that over 99.5% of bases were covered at least 1X across all samples, with approximately 88%, 65%, and 46% reaching 30X coverage in the genomic, lung metastasis and lymph node samples, respectively. Data quality and coverage uniformity were evaluated to ensure suitability for downstream variant analysis. Paired-end sequences were mapped to the human genome using Isaac aligner (iSAAC-04.18.11.09) where the reference sequence is GRCh38 (NCBI, Dec. 2013). Strelka 2.9.10 (15) was used to identify single-nucleotide variants (SNVs) and short insertions and deletions (Indels). The gvcftools, was used to generate Variant-only VCF. Variants were annotated using SnpEff v4.3 (16). SnpEff is applied to annotate the VCF file with additional databases, including ESP6500, ClinVar (17) and dbNSFP3.5 (18, 19). Somatic mutations from tumor-germline sample pairs were confirmed using VarScan (Galaxy Version 2.4.3.6) (20, 21). Variants identified by both callers were classified as confirmed. Each discrepant variant in the investigated genes was manually inspected using the Integrative Genomics Viewer (IGV). Cancer consensus genes listed in Cosmic database, genes from cancer gene list in OncoKB database and genes from oncogenic signaling pathways (14) were extracted from VCF file and presented in this paper. Results presented in this paper are achieved from have high quality reads.

## Case description

**Figure 1:**
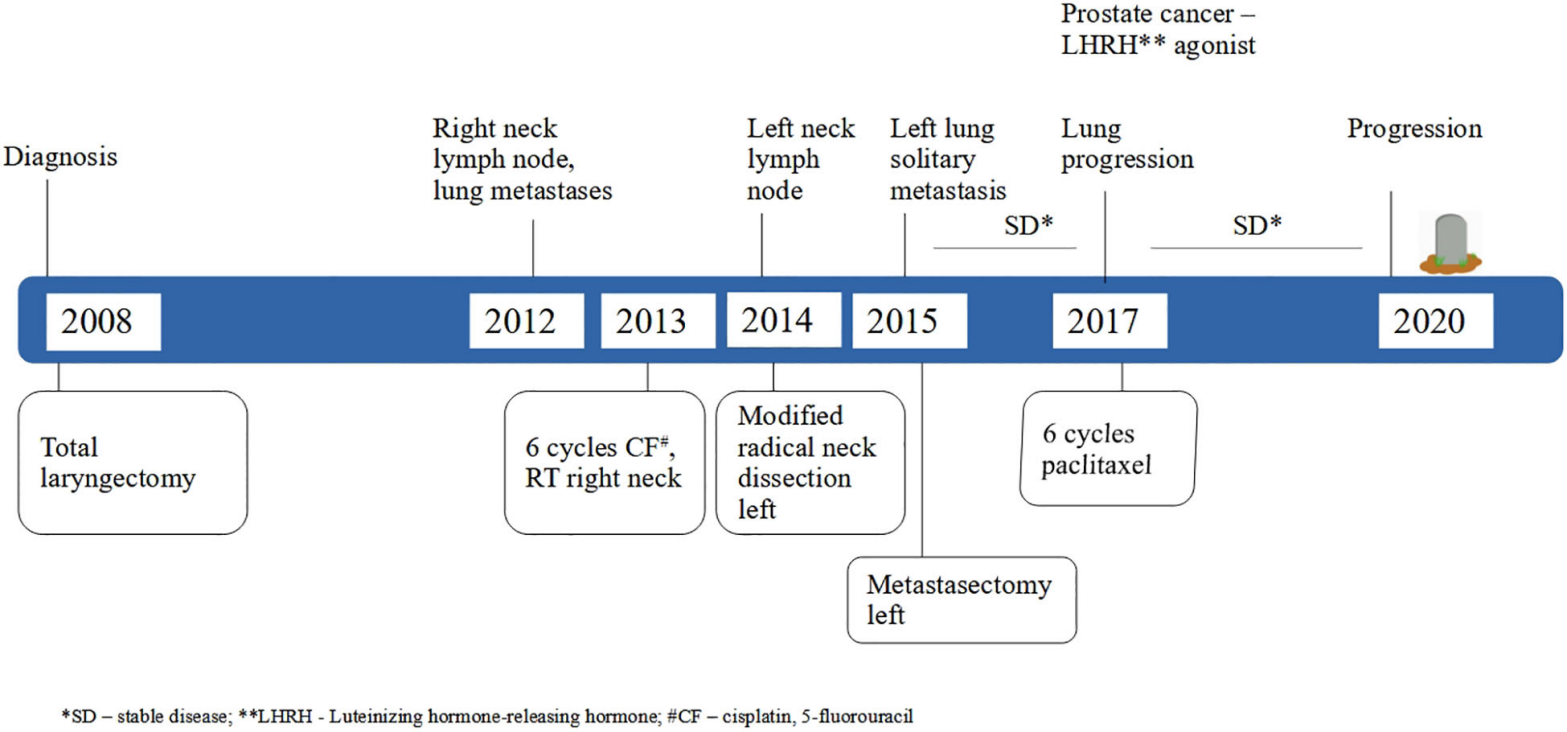
Timeline of the patient’s clinical course.

**Figure 2:**
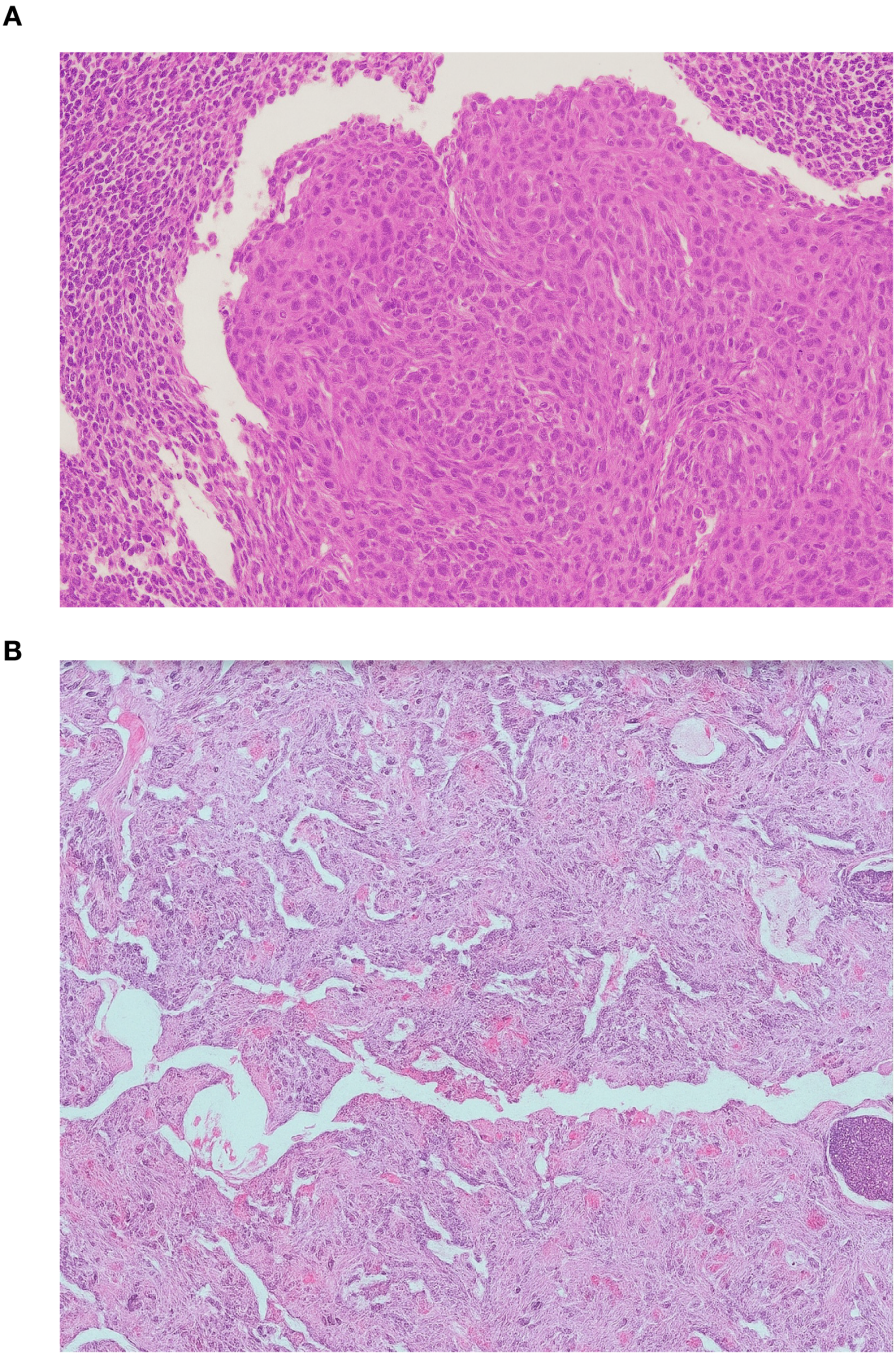
Histopathological features of metastatic laryngeal squamous cell carcinoma. **(A)** Cervical lymph node metastasis showing nests of atypical squamous epithelial cells with keratinization and intercellular bridges. **(B)** Lung metastasis displaying similar morphological characteristics confirming metastatic squamous cell carcinoma (hematoxylin and eosin stain, ×200).

A 46-year-old patient, a long-term smoker, presented to the ENT clinic in January 2008 with a two months of dysphonia. Laryngoscopic examination and neck computed tomography (CT) revealed a glottic tumor extending into the subglottic region, without evidence of cervical lymphadenopathy. The tumor was staged as cT2cN0cM0 (**Figure 1**). His medical history was notable only for previous tuberculosis. Total laryngectomy was performed. Histological analysis confirmed SCC, pT2NxMx, without adverse pathological features. The patient remained in regular follow-up without signs of recurrence until November 2012, when a palpable, painless mass was detected in the right level II neck region. Fluorine-18 fluorodeoxyglucose positron emission tomography/computed tomography (18F-FDG PET/CT) confirmed a right cervical mass measuring 2.3 x 2.2 cm, along with a solitary 1.2 cm lesion in the left upper lung lobe and a suspicious 1.5 cm nodule in the right upper lung lobe. Cytological examination of the cervical lymph node revealed SCC. From a differential diagnostic perspective, in addition to metastases from laryngeal carcinoma, a second primary lung tumor with metastasis to the cervical lymph nodes was also considered. Although metastases from a primary lung tumor are more commonly found in the supraclavicular region than in level II cervical nodes, this possibility was taken into account. Bronchoscopy did not reveal any malignant cells, and a transthoracic biopsy of the pulmonary lesion was not performed. From March to August 2013, the patient received 6 cycles of chemotherapy consisting of cisplatin 100 mg/m2 and 5-fluorouracil 1000 mg/m2 every 3 weeks. Follow-up CT imaging of the neck and chest showed stable pulmonary lesions but further enlargement of the cervical lymph node, without new metastases. In December 2013, palliative radiotherapy to the right neck was administered using 6 MV photon beams delivered by a Primus Plus linear accelerator (Siemens, Germany), equipped with an 82-leaf multileaf collimator (MLC). Total dose of 30 Gy was given in 10 fractions over two weeks. In January 2014, follow-up imaging showed significant regression of the right cervical lymph node, now 8 x 8 mm, while the right pulmonary nodule remained unchanged and the left pulmonary nodule was no longer visible. In March 2014, PET/CT revealed a solitary suspicious lymph node in the left level II neck region, without other signs of active disease. Cytology confirmed SCC. In May 2014, a modified radical neck dissection was performed, preserving the XI cranial nerve and internal jugular vein. Of 22 resected lymph nodes, one was positive for malignancy (**Figure 2A**). In October 2014, CT imaging once again identified a 14 x 12 mm nodule in the left upper lung lobe and an 8 x 8 mm necrotic lymph node in the left level II neck. PET/CT showed the pulmonary nodule as the sole site of active disease. Given the patient’s good general condition (Eastern Cooperative Oncology Group (ECOG) status 0), a pulmonary metastasectomy was performed in January 2015. Histopathological examination revealed a 1 cm tumor nodule composed of cells that were strongly positive for CK5/6 and negative for TTF-1 on immunohistochemistry. The tumor was located at one margin of the resection (**Figure 2B**). Although it is challenging to histologically distinguish a metastatic laryngeal SCC in the lung from a primary pulmonary SCC, as both share similar morphological features, the clinical history, radiologic findings, and immunohistochemistry in this case supported the conclusion that the pulmonary lesions represented metastases from the laryngeal carcinoma. No clinical or radiological evidence of recurrence was observed until February 2017, when PET/CT detected progression in the left lung. An incidental focal radiotracer uptake in the prostate prompted biopsy, confirming prostate adenocarcinoma with a Gleason score 4 + 4 = 8. At diagnosis, the prostate-specific antigen (PSA) level was 6.78 μg/l. Androgen deprivation therapy with luteinizing hormone-releasing hormone (LHRH) agonists was initiated in April 2017. According to the available literature, there is no known etiological or molecular link between laryngeal SCC and prostate adenocarcinoma. Thus, the simultaneous occurrence of these two tumor types in this patient likely reflected the independent development of two distinct malignancies with different risk factors. Due to progression of pulmonary metastases, the patient received 6 cycles of paclitaxel 175 mg/2 every 3 weeks from May to September 2017. Imaging showed stable disease with no significant progression. Prostate cancer remained well-controlled with PSA nadir < 0.01μg/l by January 2018. The patient tolerated the treatment well, experiencing no adverse effects other than occasional fatigue and showed good adherence to the therapy schedule. No dose modifications of chemotherapy were required, likely due to the relatively short duration of therapy, which was never longer than six consecutive months and included treatment-free intervals. The patient maintained a good quality of life until early 2018 and remained generally optimistic, although some concern arose with disease relapse. Despite radiological stability of the lung metastases and consistently low PSA levels, the patient’s general condition gradually declined starting in early 2018. Regular follow-up was continued with CT imaging, laboratory tests, and clinical examinations every four months. In November 2020, CT imaging revealed widespread metastatic disease, including new lesions in the lung, liver, cervical, mediastinal and retroperitoneal lymph nodes as well as osteolytic bone metastases. PSA had risen to 6.70 μg/l and the patient’s performance status deteriorated to ECOG 4. He died on December 14, 2020, eight years following the diagnosis of metastatic laryngeal disease.

We performed next generation sequencing on tumor (lymph node and lung) and germline DNA from described patient. In this paper we analyzed only single nucleotide polymorphisms (SNPs) and small insertions and deletions (INDELs). The putative impact of genetic variants was categorized into four predefined impact levels based on their predicted effect on protein function, as determined by SnpEff. High-impact variants cause disruptive changes such as frameshifts, stop codon gains or losses, or splice site disruptions that are likely to severely affect protein function. Moderate-impact variants include non-synonymous changes such as amino acid substitutions or in-frame insertions/deletions that may alter protein function but are less likely to be fully disruptive. Low-impact variants are typically synonymous changes that do not alter the amino acid sequence and are expected to have minimal or no effect on protein function. MODIFIER-impact variants are generally located in non-coding regions or upstream/downstream regions where the functional consequences are less clear or indirect. Highimpact variants were further cross-checked against population frequency data from gnomAD, and for variants with low population frequency, pathogenicity was assessed using online resources including MutationMapper at cBioPortal and in silico predictors available on gnomAD.

### Somatic mutations in oncogenic signaling pathways

Since genetic alterations in signaling pathways that control cell-cycle progression, apoptosis, and cell growth are common hallmarks of cancer we analyzed oncogenic signaling pathways (cell cycle, Hippo, Myc, Notch, Nrf2, PI-3-Kinase/Akt, RTK-RAS, TGFβ signaling, p53 and β-catenin/WNT) comprising a total of 266 genes (14), in both tumor and germline DNA. **Table 1** summarizes the mutated genes identified in the lymph node and lung metastases, along with the corresponding mutation types. Somatic mutations were found in all 10 oncogenic signaling pathways. Lymph node sample harbored 35 mutations across 19 genes, including 11 mutations in EIF4EBP1 (Eukaryotic translation initiation factor 4E-binding protein 1). The lung metastasis sample contained 39 mutations in 31 genes with 3 mutations in CTBP2 (C-Terminal Binding Protein 2). Two mutations, one in *MXI1* and one in *PIK3R2*, belonging to the MYC and PI3K pathways, respectively, were shared between both tumor samples. We compared our data with a curated set of non-redundant studies in cBioPortal (22–24) for cancer genomics (80085 samples) and found four identical mutations but none of them were observed in HNC samples within the database. Putative impact of all but one mutation was unknown, low or moderate.

**Table 1:** Somatic mutation distribution in signal pathway genes.

Signal pathway	Gene	Samples			
		Lymph nodes		Lung metastasis	
Cell Cycle	CDK2			1	
	CDKN2C	1			
HIPPO	DCHS2			1	
	HIPK2	1		1	
	LIMD1			2	
	MOB1B			1	1
	PTPN14			1	
	TAOK1			1	1
	TAOK2			1	
	WTIP	2		1	
	WWC1	1			
MYC	MAX			2	
	MXI1	1		2	
NOTCH	CNTN6			1	
	CTBP2			3	
	DLK1			1	
	DLL3	1		1	
	HES2			1	
	HEY1			1	
	HEYL			1	
	MAML3			1	
	NUMB			1	
	PSENEN			1	
	FBXW7			1	
	ADAM17	1			
NRF2	CUL3			1	
	NFE2L2	2			
PI3K	AKT1S1			1	
	EIF4EBP1	11			
	PIK3R2	2		1	
	TSC1	1			
RTK RAS	IRS2	1			
	KSR2			1	
	PIN1			1	
TFK-β	SMAD2			1	
TP53	MDM4	3			
	TP53	1			
WNT	AMER1	1			
	FZD1	1	1		
	FZD3			1	
	LZTR1			1	
	TLE3			1	
	WNT4	1			
	DKK1	1			

5_prime_UTR_variant

5_prime_UTR_premature_start_codon_gain_variant

synonymous_variant

missense_variant

missense_variant&splice_region_variant

splice_region_variant&non_coding_transcript_exon_variant

3_prime_UTR_variant

### Germline variants in oncogenic signaling pathways

In Germline DNA, we identified 21 high impact mutations in genes from the analyzed oncogenic signaling pathways, all of which have been previously reported (**Supplementary Table S1**). Notably, only three of the 21 variants had a population frequency below 1%, making them potentially deleterious (25). One such rare, high impact variant, rs774201781, is an in-frame deletion or intronic variant in the *MLL3* gene, found in the germline sample in heterozygous form. This variant has a population frequency of 0.0335% in individuals of European ancestry (26), and has recently been associated with Lynch syndrome in a Chinese family (27). Another high impact mutation is a synonymous mutation in LGR5 which is characterized as a structural interaction variant (rs140637499). Although silent mutations were historically considered functionally irrelevant, emerging evidence suggests they may influence transcription and translation in various ways (28). The third high impact germline mutation was found in TP53, gene not only involved in one of the analyzed signaling pathways but also among the most extensively studied genes in cancer biology.

### Somatic and germline variants in consensus cancer genes

Furthermore, we searched somatic and germline variants in genes listed as cancer consensus genes in the COSMIC database and in the OncoKB cancer gene database. In germline DNA, 23 high-impact mutations were identified but most of these were common variants according to gnomAD population frequency data (26). Only three were rare: two frameshift mutations in *SERPINB3* (rs60533853 and rs201374310), each with a population frequency of ~1%, and one rare variant in *ASMTL* (chrX:1418076), present in only four samples in the gnomAD database. According to the CADD in silico predictor, this variant is likely deleterious (29). In tumor DNA, a high-impact mutation in a cancer consensus gene was identified in the *SH2B3* gene in the lymph node sample. This T>C transition at position 12:111447546 is a splice donor variant found in a heterozygous state and is predicted to be moderately pathogenic by CADD.

The lung metastasis sample contained high-impact mutations in four cancer-related genes. Among them, variants in *RAD51B* (rs751355274), *IRF8* (rs903202), and *SYK* (rs35758162) were found to be common in the general population (e.g. gnomAD database) and therefore are unlikely to be deleterious. One rare mutation was detected in FOLH1 (rs79155991) reported in six samples in cBioPortal but not in any HNC cases. ClinVar classifies it as benign. Another mutation, in *FGFR3* (rs587777857), was identified in one colon adenocarcinoma sample in cBioPortal and is predicted to be moderately benign by CADD.

According to Malone and Siu (30), the most frequently mutated genes in head and neck cancers include *TP53*, *CDKN2A*, *PIK3CA*, *KMT2D*, *NOTCH1*, and *FAT1*. **Supplementary Figure S1** shows the mutation positions in these genes across germline, lymph node, and lung metastasis samples.

## Discussion

Despite recent treatment advances in R/M HNSCC, survival has lagged behind other tumor sites, with only 15–23.9% of patients alive at five years (5–8, 31). Furthermore, previous studies have demonstrated considerable variability in treatment and survival outcomes for recurrent/metastatic HNSCC in real-world settings (32), largely due to regulatory and reimbursement limitations, as well as restricted access to certain therapies.

At the time of our patient’s metastatic diagnosis in 2012, cetuximab was not available for advanced HNSCC treatment in Croatia, nor were immunotherapy or advanced radiotherapy techniques such as stereotactic radiotherapy. Consequently, the patient received only standard chemotherapy, supplemented by metastasectomy and palliative radiation when appropriate.

Despite the suboptimal systemic treatment, several clinical factors likely contributed to the patient’s prolonged survival. Notably, the patient presented with oligometastatic disease at the time of R/M diagnosis, an intermediate disease state between localized and widespread metastases, associated with improved survival outcomes (4). Additionally, metastatic disease was diagnosed four years after initial treatment of the primary tumor, a longer interval than typically expected (33). However, despite the early stage at initial diagnosis and generally low relapse risk, the patient still experienced disease recurrence.

Whole genome sequencing data provided valuable insights into genomic alterations associated with tumor samples from lymph node and lung metastases in comparison to the patient’s germline DNA. Focusing on SNPs and small INDELs we concentrated our analysis on key oncogenic signaling pathways known to play critical roles in cancer biology (14). In line with previous reports, mutations were detected across all ten analyzed pathways, reinforcing the ubiquity of such alterations in tumorigenesis.

The lymph node sample exhibited a total of 35 mutations across 19 genes with a striking prevalence of 11 mutations in the EIF4EBP1 gene. It plays a crucial role in the regulation of protein synthesis, which can impact various cellular processes, including cell growth and proliferation. In HNC, EIF4EBP1 is particularly relevant due to its inhibition of eIF4E, a key translation initiation factor. Phosphorylation of EIF4EBP1 releases eIF4E, facilitating oncogenic protein synthesis. In cancer, especially HNSCC, this mechanism can contribute to the overexpression of oncogenic proteins (34). Huang et al. (35) suggested that the expression of eIF4E and p-4EBP1 should be considered as predictive biomarkers for the HNSCC patients. All 11 mutations in this gene were located in the 3′ untranslated region (3′ UTR), which may affect post-transcriptional regulation.

In the lung metastasis sample the most frequently mutated gene was CTBP2 with four mutations also located in the 3′ UTR. *CTBP2* functions as a transcriptional corepressor that interacts with transcription factors and chromatin-modifying complexes, primarily acting to repress tumor suppressor genes and those involved in differentiation, apoptosis, and cell cycle regulation. While no studies have specifically focused on EIF4EBP1 or CTPB2 3’ UTR variants in HNSCC, the general role of 3’ UTR variants in cancer suggests they could affect the function of these genes. Variants in the 3’ UTR of other genes have been shown to disrupt regulatory elements such as microRNA binding sites, alter transcript stability, or change mRNA localization, potentially leading to altered protein expression or function. Recent findings also suggest that 3′ UTR variants can influence mRNA abundance post-transcriptionally (36). Overall, mutations in cancer signaling pathways were predominantly located in the 3′ UTR and included other splice-related variants.

Analysis of the germline genome revealed 21 high-impact mutations in cancer signaling pathway genes. Only three of these variants were rare and potentially deleterious. One such mutation is an in-frame deletion in *MAML3*, a transcriptional coactivator involved in the Notch signaling pathway (37). This variant has been associated with Lynch syndrome in a recent study (27), underscoring the need to monitor rare germline variants linked to hereditary cancer risk. While functional validation is required, such findings support the value of integrating germline data into cancer profiling. Although direct connections between germline and somatic mutations could not be established in this case, growing evidence suggests that the interplay between germline and somatic variants contributes significantly to tumor development (38).

Comprehensive cancer genome sequencing studies have shown that most cancers contain a small number of frequently mutated well-known oncogenes and tumor suppressors (such as p53 and PIK3CA), alongside a larger pool of less frequent, unique mutations. Every individual carries numerous germline variants, both common and rare, that may affect gene function, influence disease susceptibility, and contribute to the heritability of cancer. While a subset of cancers can be attributed to high penetrance germline mutations, the majority arise in the absence of such variants (39). In our analysis of cancer consensus genes from the Cosmic and OncoKB databases, we identified several high-impact mutations. Most were common variants, but rare mutations were found in *SERPINB3* and *ASMTL*, meriting further investigation. High impact somatic mutations were detected in the lymph node sample in *MSH2*, *OMD*, and *SH2B3*. These mutations are predicted to be pathogenic, although they are not well-characterized in current databases. In contrast, the lung metastasis sample exhibited high impact mutations in *RAD51B*, *IRF8*, and *SYK*, all of which are common in the general population and thus less likely to be deleterious. Notably, methylation of *RAD51B* has been associated with immune checkpoint expression and inflammatory signatures in HNSCC (40), and *SYK* is recognized as a downstream effector of EGFR signaling implicated in HNSCC development (35). A rare mutation was also identified in *FOLH1*, though it is currently classified as benign in ClinVar.

We also compared germline and somatic mutations in genes most frequently altered in HNSCC. Interestingly, these genes harbored more germline than somatic mutations. Somatic TP53 mutation C176S occurs within its DNA-binding domain (41). This variant is associated with increased protein stability (42), it impairs p21 induction and disrupts disulfide-dependent complex formation, leading to predicted loss of *TP53* function (43). The germline variant P72R is a common polymorphism located within the regions of *TP53* that interact with CCAR2, HRMT1L2, and WWOX (44). Previous studies have reported a lower frequency of *TP53* mutations in metastases compared to the primary HNC (45, 46).

Although most research has focused on identifying mutations that lead to cancer, studies on long-term cancer survivors are much rarer. Bowell et al. (47) have found that specific combinations of germline and somatic gene alterations, tumor cell phenotypes, and differential immune responses appear to contribute to long-term survival in high-grade serous ovarian cancers. Similar studies in log-term HNSCC survivors could provide insights into individual risks profiles and tumor behaviors, and the present case would be a valuable example to include in such an investigation.

## Conclusion

This case highlights the rare but significant occurrence of long-term survival in a patient with metastatic laryngeal SCC treated solely with conventional chemotherapy, surgery, and palliative radiotherapy. Despite the absence of targeted therapy or immunotherapy, the patient achieved an exceptional overall survival of eight years from the onset of metastatic disease. Our genomic analysis revealed diverse somatic and germline alterations across multiple oncogenic signaling pathways, including frequent 3′ UTR mutations in EIF4EBP1 and CTBP2, and rare germline mutations with potential deleterious effects. These findings support the hypothesis that unique molecular features may underlie unusually favorable outcomes in a subset of patients with R/M HNSCC. Further research is warranted to investigate the functional relevance of these alterations and to explore whether they may serve as biomarkers for prognosis or treatment de-escalation in selected individuals.

## Data Availability

The datasets presented in this article are not readily available beceause they are part of ongoing research. Requests to access the datasets should be directed to MPS (mpericic@inantro.hr).
