# Supplementary material for: Genomic landscape of a long-term surviving patient with metastatic cancer of the larynx: a case report
Source: Front Oncol. 2025 Dec 2;15:1691802. doi: 10.3389/fonc.2025.1691802 (PMC12705357; doi:10.3389/fonc.2025.1691802)
Supplement: Supplementary file 1 [file DataSheet1.docx]

**Supplementary material**


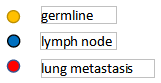


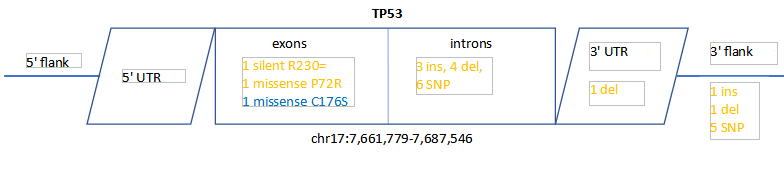


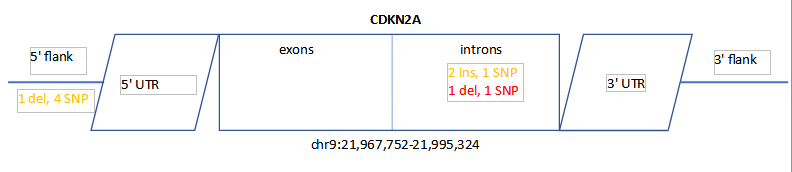


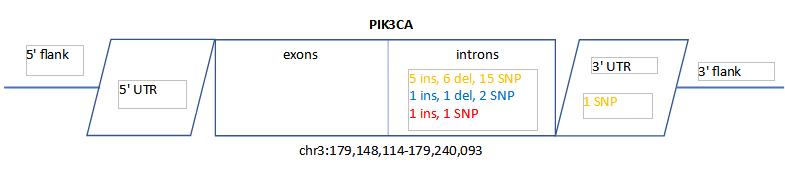


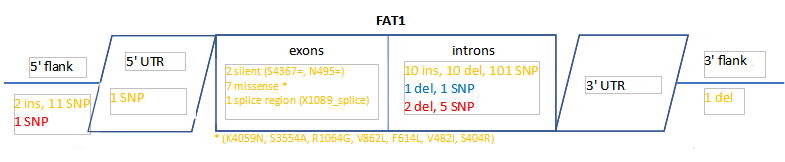

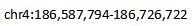


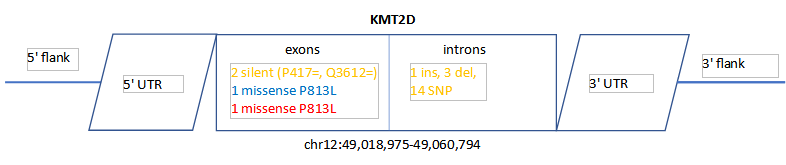


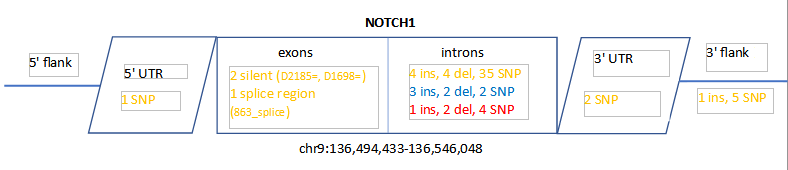


Figure S1. The mutation positions in TP53, CDKN2A, PIK3CA, KMT2D, NOTCH1 and FAT1 genes across germline, lymph node and lung metastasis

Table S1. High-impact germline variants in oncogenic signalling pathways
